# First Line Androgen Deprivation Therapy Duration Is Associated with the Efficacy of Abiraterone Acetate Treated Metastatic Castration-Resistant Prostate Cancer after Docetaxel

**DOI:** 10.3389/fphar.2017.00055

**Published:** 2017-02-13

**Authors:** Jian-Ri Li, Shian-Shiang Wang, Cheng-Kuang Yang, Chuan-Su Chen, Hao-Chung Ho, Kun-Yuan Chiu, Chi-Feng Hung, Chen-Li Cheng, Chi-Rei Yang, Cheng-Che Chen, Shu-Chi Wang, Chia-Yen Lin, Yen-Chuan Ou

**Affiliations:** ^1^Division of Urology, Department of Surgery, Taichung Veterans General HospitalTaichung, Taiwan; ^2^Institute of Medicine, Chung Shan Medical UniversityTaichung, Taiwan; ^3^Department of Medicine and Nursing, Hungkuang UniversityTaichung, Taiwan; ^4^Department of Applied Chemistry, National Chi Nan UniversityNantou, Taiwan; ^5^Department of Urology, China Medical University HospitalTaichung, Taiwan; ^6^Department of Medical Research, Taichung Veterans General HospitalTaichung, Taiwan

**Keywords:** abiraterone acetate, androgen deprivation therapy, castration-resistant prostate cancer, docetaxel, PSA

## Abstract

**Introduction:** We performed a chart review study in our castration-resistant prostate cancer (CRPC) patients who received Abiraterone acetate (AA) treatment after docetaxel and identified clinical markers which can predict treatment outcome.

**Materials and Methods**: From 2012 to 2016, 64 patients who received docetaxel after CRPC followed by AA treatment were included. Clinical parameters were recorded and analysis was performed to identify associations between pre-treatment variables and treatment outcome.

**Results**: Thirty three patients (51.6%) achieved a decrease in PSA of 50%. The median PSA progression-free survival and overall survival in the total cohort of 64 patients were 6.6 and 24 months, respectively. Adverse events (AEs) in all grades developed in 35.9% (23/64) patients and mostly were grade 1 or 2. The most common AEs were gastric upset, hypokalemia and elevated liver function tests. Of the eight variables analyzed, first line androgen deprivation therapy (ADT) duration showed positive association to progression free survival (HR 0.98, 95% CI [0.96–0.99], *p* = 0.012) and overall survival (HR 0.97, 95% CI [0.94–0.99], *p* = 0.019). Pre-AA PSA and PSA progression ratio showed negative association only to progression free survival (HR 1.0, 95% CI [1.000–1.002], *p* = 0.025, HR 1.01, 95% CI [1.00–1.01], *p* < 0.001, respectively).

**Conclusion**: First line ADT duration was positively associated with AA treatment efficacy in progression free survival and overall survival. It can be used as a pre-treatment predictor.

## Introduction

Abiraterone acetate (AA) has been shown to be effective in prolonging progression-free survival and overall survival in metastatic castration-resistant prostate cancer (MCRPC) after docetaxel chemotherapy (de Bono et al., 2011). Although the recent COU-AA-302 (Cougar Biotechnology, AA) study showed a clinical benefit of AA in a chemo-naïve setting, it still have more clinical applications using the post-chemotherapy model for advanced prostate cancer patients in Asian countries. In the subgroup analysis of the COU-AA-301 trial, AA showed an equivalent efficacy in the Asian group compared with data from Western countries with progression-free survival (Fizazi et al., 2012; Kwak et al., 2014). Various parameters were used for prediction of patient outcome during AA treatment, but still controversial (Azad et al., 2015; Houede et al., 2015; Xu et al., 2015; Bellmunt et al., 2016; Chi et al., 2016; Facchini et al., 2016; Rescigno et al., 2016). Herein, we conducted a clinical investigation of AA in CRPC patients after chemotherapy and validated several clinical factors correlated with progression-free survival and overall survival before AA treatment.

## Materials and Methods

### Patients

This was a retrospective chart-review study which analyzed metastatic castration prostate cancer patients after chemotherapy using AA between 2012 and 2016. Of the 71 consecutive patients, 7 were excluded because of incomplete data or loss of follow-up and were also excluded. All included patients received informed consent before treatment according to the certification of the institute review board of Taichung Veterans General Hospital, number CE13240A-2. The rest 64 patients all had bone metastases and were included in this analysis. All patients received AA 1000 mg with prednisolone 5 mg or 10 mg per day. Patients received proper pain control with opioid medication or palliative radiation therapy according to clinical requirements.

### Study Assessment

The primary patient characteristics were age at start of AA treatment, serum PSA level at MCRPC, PSA at chemotherapy, PSA at AA treatment, first-line chemotherapy cycles, second-line cycles, first-line androgen deprivation therapy (ADT) duration, chemotherapy duration, PSA progression velocity before AA, PSA progression ratio before AA treatment, follow-up duration, progression-free survival of AA treatment, overall survival of AA treatment and survival status. Maximal effect period was recorded as the time period required to the lowest PSA level after AA treatment. The adverse events (AEs) developed during treatment were also recorded.

The first-line ADT duration was defined as the months between the date of ADT beginning and the date of CRPC. First-line ADT included surgical castration (orchiectomy) or medical castration using LH-RH agonists or antagonists. The chemotherapy cycles were defined according to the standard 3-week docetaxel treatment. In our practice, 2-week and 4-week courses of chemotherapy were performed. We transferred the 2-week cycles into standard 3-week cycle counts. PSA progression was defined according to the Prostate Cancer Working Group second publication (PCWG2) criteria (Scher et al., 2008). Some patients also received bone scan and evaluation symptoms to better define their progression. Although the clinical management did not completely match the definition criteria, such as delayed or early chemotherapy at the time of PSA progression, we still recorded the date of treatment. Chemotherapy duration was defined as the months between the date of AA treatment beginning and the date of chemotherapy beginning. PSA progression velocity before AA was defined as the PSA change between the pre-AA treatment and the CRPC divided by the time interval. PSA progression ratio was defined as PSA before AA treatment divided by the CRPC PSA level.

### Statistical Analysis

The differences between continuous values were analyzed by Mann–Whitney *U* test and Fisher’s exact test *t*-test for continuous variables. χ^2^ test was used for categorical variables. The progression-free survival and overall survival curves were plotted using the Kaplan–Meier method with statistical significance examined by the log-rank test. Univariate and multivariate Cox hazard regression was used to estimate the hazard ratio (HR) and 95% confidence interval (CI) for association between variables and progression-free and overall survival. All statistical analyses were performed using SAS software version 9.2 (SAS Institute, Inc., Cary, NC, USA). A *p*-value of < 0.05 was considered statistically significant.

## Result

Thirty-three patients (51.6%, 33/64) met the criteria of PSA decline greater than 50% after AA treatment. **Table 1** shows the basic characteristics among all 64 patients. During the median 7.9 months follow-up period, 43 patients (67.2%) were still alive. The median first line ADT duration was 26.9 months and the median fist line chemotherapy used was seven cycles. Twenty-five patients (39.1%) received second line chemotherapy with cabazitaxel with a median of four cycles. The overall chemotherapy duration before AA treatment was 17.1 months.

**Table 1:** Basic characteristics of patients receiving Abiraterone acetate treatment.

	Median	Range
Age at AA treatment (years; *n* = 64)	72	49–89
Received radical prostatectomy (*n*, %)	15	23.4%
Start 1st line ADT age (months; *n* = 64)	66.0	46–87
1st line ADT duration (months; *n* = 63)	26.9	2.3–144.5
CRPC PSA (*n* = 63)	5.3	2–354
1st line chemo cycles (*n* = 63)	7.0	1–33
2nd line chemo cycles (*n* = 25)	4.0	3–12
Chemotherapy period (months; *n* = 63)	17.1	2.7–71
Pre-AA PSA (*n* = 64)	42.7	4–1941
PSA response (*n*, %)	33	51.6%
PSA decline % (*n* = 64)	80.3	-269–99.9
Total follow-up period (months; *n* = 64)	7.9	0.9–46.5
Maximal effect period (months; *n* = 37)	3.6	0.5–11.7
Progress-free survival (months; *n* = 64)	4.1	0.8–25.2
PSA velocity (PSA/months; *n* = 63)	1.0	-23.2–78.1
PSA ratio (*n* = 63)	6.5	0.3–447.3
Survival (*n*, %)		
Alive	43	67.2%
Death	21	32.8%


*AA, abiraterone acetate; ADT, androgen deprivation therapy; CRPC, castration-resistant prostate cancer. PSA decline, (Pre-AA PSA subtract Lowest PSA after AA) divided by Pre-AA PSA. PSA velocity before AA, (Pre-AA PSA subtract CRPC PSA) divided by (Date start AA subtract Date CRPC). PSA ratio, Pre-AA PSA divided by CRPC PSA.*

**FIGURE 1:**
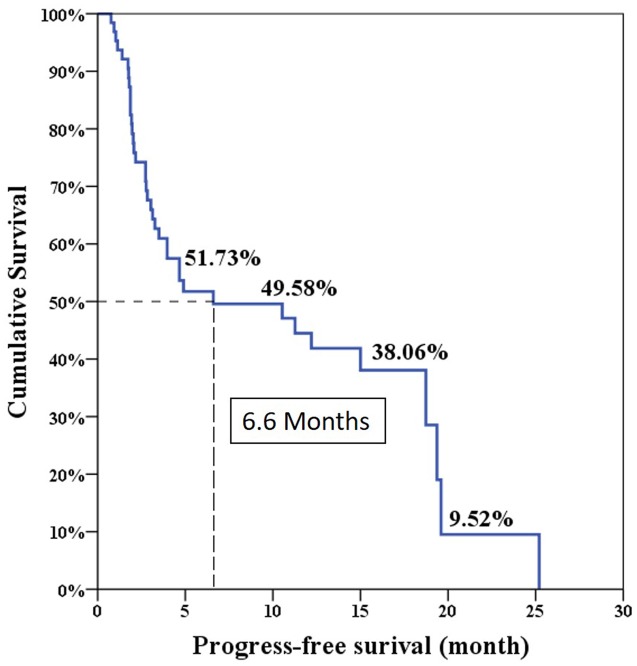
**Progression-free survival in total 64 patients**.

**FIGURE 2:**
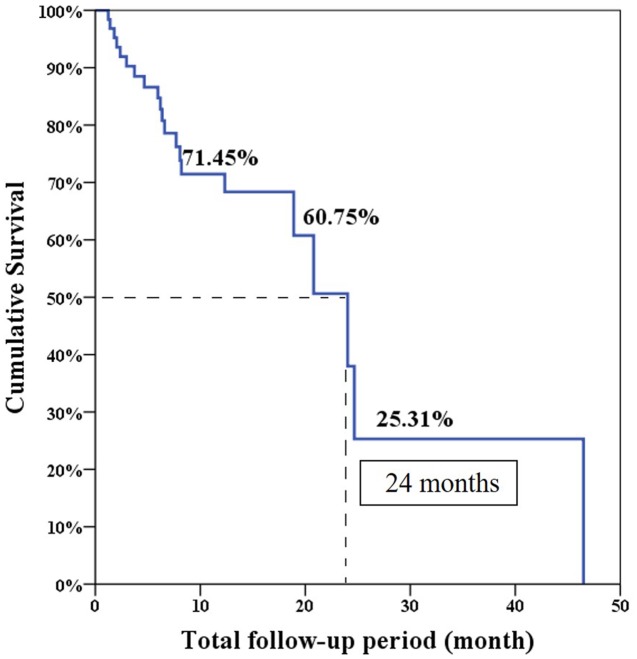
**Overall survival in total 64 patients**.

The median progression free survival expected using the Log-rank test was 6.6 months and the overall survival was 24 months (**Figures 1** and **2**).

**Table 2** shows the development of AEs during AA treatment. The development percentage of grade 1/2 and grade 3/4 AE was 31.3% (20/64) and 4.7% (3/64), respectively. However, one case of hyperkalemia developed in the PSA non-responder group and resulted in patient death. Two patients experienced elevated glutamate oxaloacetate transaminase (GOP) and glutamate pyruvate transaminase (GPT). They received temporary suspension of AA; one of them recovered after 1 week and was kept on treatment without tapering dosage and the other had PSA progression.

**Table 2:** Adverse events during abiraterone acetate treatment.

	Patients (*n* = 64)	
	
	Grade 1/2	Grade 3/4
Subjects with AE, *n* (%)	20 (31.3)	3 (4.7)
General AE, *n* (%)		
Diarrhea	1 (1.6)	0
Hypokalemia	3 (4.7)	0
Hyperkalemia	1 (1.6)	1 (1.6)
Fatigue	2 (3.1)	0
Vertigo	1 (1.6)	0
Stasis dermatitis	2 (3.1)	0
Leg edema	4 (6.3)	0
Puffing face	1 (1.6)	0
Gastric upset	4 (6.3)	0
Elevated liver function	1 (1.6)	2 (3.1)


In univariate Cox regression analysis, first-line ADT duration showed a positive association to better progression free survival; pre-AA PSA, and PSA progression ratio showed a negative association to longer progression free survival. After adjustment, all three factors were considered predictors of PSA progression before AA treatment (**Table 3**). In the analysis of overall survival, only first line ADT duration reached the statistical significance (*p* = 0.019) (**Table 4**).

**Table 3:** Predictive variables of progression-free survival.

	Univariate analysis			Multivariate analysis		
		
	HR	95% CI	*P*-value	HR	95% CI	*P-*value
Age at AA treatment	0.99	(0.95–1.03)	0.476			
1st line ADT duration (months)	0.98	(0.96–0.99)	0.011^∗^	0.98	(0.96–0.99)	0.012^∗a^
CRPC PSA	1.00	(1.00–1.01)	0.163			
Pre-chemo PSA	1.00	(1.00–1.00)	0.544			
1st line chemo cycles	0.99	(0.94–1.03)	0.597			
Pre-AA PSA	1.00	(1.00–1.00)	0.023^∗^	1.00	(1.000–1.002)	0.025^∗a^
PSA velocity before AA (PSA/month)	1.01	(1.00–1.02)	0.138			
PSA progression ratio	1.00	(1.00–1.01)	0.001^∗^	1.01	(1.00–1.01)	<0.001^∗b^


*Cox regression. HR, hazard ratio. ^a^Adjusted for 1st line ADT duration (months), Pre-AA PSA, ^b^Adjusted for 1st line ADT duration (months), PSA progression ratio. ^∗^*P* < 0.05.*

**Table 4:** Predictive variables of overall survival.

	Univariate analysis		
	
	HR	95% CI	*p-*value
Age at AA treatment	1.01	(0.96–1.07)	0.695
1st line ADT duration (months)	0.97	(0.94–0.99)	0.019^∗^
CRPC PSA	1.00	(0.99–1.01)	0.924
PRE-chemo PSA	1.00	(1.00–1.01)	0.428
1st line chemo cycles	0.96	(0.89–1.03)	0.289
Chemo period (month)	0.98	(0.94–1.02)	0.255
Pre-AA PSA	1.00	(1.00–1.00)	0.186
PSA velocity before AA (PSA/month)	1.01	(1.00–1.03)	0.069
PSA progression ratio	1.00	(1.00–1.01)	0.270


*Cox regression. HR, hazard ratio. ^a^Adjusted for 1st line ADT duration (month).^∗^*p* < 0.05.*

## Discussion

Our study provided the evidence that clinical, non-laboratory parameters can also predict patient outcome using AA treatment in post-docetaxel MCRPC. Several clinical factors had been used as outcome predictors. Chi et al. (2016) used database from COU-AA-301 study and found serum lactate dehydrogenase (LDH), Eastern Cooperative Oncology Group Performance Status (ECOG PS), liver metastases, serum albumin, serum alkaline phosphatase (ALP) and first line ADT duration could act as a cumulative scoring system to predict AA treatment efficacy. Our data corresponded to their finding on the ADT treatment duration. However, lack of most of lab blood tests or image study results makes our finding limited. Facchini et al. (2016) reported early PSA response could predict the overall survival after AA treatment which was a post-treatment prediction. Our study is the first to show pre-treatment clinical markers such as first-line ADT duration, pre-AA PSA level, and PSA progression ratio may correspond to progression-free survival and may serve as a simpler tool for predicting AA treatment outcome.

Molecular factors such as androgen-receptor splice variant seven messenger RNA (AR-V7) has been well studied in the prediction of the efficacy of second-line hormone therapy, especially AA and enzalutamide (Antonarakis et al., 2014). AR-V7 not only explained the possible drug-resistant mechanism, it may have value as a predictive tool before second-line hormone therapy.

The overall PSA response rate was 51.6% (33/64) in our study which was better than the 29.5% response rate found in the COU-AA-301 trial. Our data were similar to the findings of a previous Korean and Taiwanese small group study which showed a PSA response rate of 43% (Kwak et al., 2014). However, our results showed the median PSA progression-free survival was only 6.6 months which was shorter than the duration of 8.5 months in the COU-AA-301 trial. This is because the definition of treatment failure in our series was only based on PSA progression. Interestingly, our median overall survival was 24 months which was longer than the duration of 15.8 months in the COU-AA-301 trial. This is the result of some patients who experienced longer overall survival (longest follow-up 46.5 months) in our database which made survival prediction right shift.

The median time to maximal PSA response was 3.6 months which implied most patients who were sensitive to AA treatment would experience early PSA response in about 3 months period. This finding corresponded to Facchini’s results in the post-treatment observation. However, one patient did not reach lowest PSA decline until 9.3 months. This patient did have early PSA decline about 55% after 6 months, he received prednisolone shift to dexamethasone and experienced more PSA decline to 90%. Similar findings were reported by Lorente et al. (2014).

Our data showed most patients tolerated AA well and only 4.7% (3/64) had grade 3/4 AEs. One of them developed severe hyperkalemia and died of shock. The precise etiology was unknown but a latent infection which induced septic shock and acute renal failure was suspected. Some patients developed leg and facial edema and symptoms similar to metabolic syndrome in previous reports (Logothetis et al., 2012).

The treatment strategy of MCRPC has been discussed in the literature in recent years (Sonpavde et al., 2015; van Dodewaard-de Jong et al., 2015; van Soest et al., 2015). In our study, 29.7% (19/64) patients received cabazitaxel before AA in the early period. After AA was insurance reimbursed, no patients received cabazitaxel before AA. Ten patients received docetaxel after AA failure which showed no obvious survival benefit as previous reports (Zhang et al., 2015).

There were several limitations in our study. First, this is a retrospective chart review study. PSA check-up schedules and many variables were not well controlled. Second, PSA was the only parameter to define treatment outcome in our study. It is now considered PCWG3 consensus as a recommended evaluations in CRPC studies (Scher et al., 2016). There would be outcome variations in such different standards. Third, six patients in the PSA responder group received prednisolone shift to dexamethasone after PSA mild elevation. All of them experienced PSA decline after shifting and it was regarded as treatment continuing in our study. Although this design might influence progression free survival, there should be no differences in the overall survival comparison. Fourth, our sample size was too small and the HR seemed no such great impact even reaching statistical significance.

## Conclusion

Our real-world patient data showed that pre-treatment predictors of AA in PSA progression-free survival included first-line ADT duration, pre-AA PSA, and PSA progression ratio. For overall survival, first-line ADT duration can also serve as a pre-treatment predictor. PSA response itself can be applied as post-treatment predictors for PSA progression-free survival and overall survival after prescription.

## Ethics Statement

This is a retrospective case analysis in a single institute.

## Author Contributions

Case collection: J-RL, S-SW, C-KY, C-SC, H-CH, K-YC, C-FH, C-LC, C-RY, C-CC, S-CW, C-YL, Y-CO. Manuscript writing: J-RL.

## Conflict of Interest Statement

The authors declare that the research was conducted in the absence of any commercial or financial relationships that could be construed as a potential conflict of interest.
